# Tumor Suppressors in Chronic Lymphocytic Leukemia: From Lost Partners to Active Targets

**DOI:** 10.3390/cancers12030629

**Published:** 2020-03-09

**Authors:** Giacomo Andreani, Giovanna Carrà, Marcello Francesco Lingua, Beatrice Maffeo, Mara Brancaccio, Riccardo Taulli, Alessandro Morotti

**Affiliations:** 1 Department of Clinical and Biological Sciences, University of Torino, 10043 Orbassano, Italy; giacomo.andreani@unito.it (G.A.); giovanna.carra@unito.it (G.C.); beatrice.maffeo@edu.unito.it (B.M.); 2 Department of Oncology, University of Torino, 10043 Orbassano, Italy; marcello.lingua@edu.unito.it (M.F.L.); riccardo.taulli@unito.it (R.T.); 3 Department of Molecular Biotechnology and Health Sciences, University of Torino, 10126 Turin, Italy; mara.brancaccio@unito.it

**Keywords:** chronic lymphocytic leukemia, tumor suppressors, mutations, deletions, epigenetics, miRNA, p53, PTEN

## Abstract

Tumor suppressors play an important role in cancer pathogenesis and in the modulation of resistance to treatments. Loss of function of the proteins encoded by tumor suppressors, through genomic inactivation of the gene, disable all the controls that balance growth, survival, and apoptosis, promoting cancer transformation. Parallel to genetic impairments, tumor suppressor products may also be functionally inactivated in the absence of mutations/deletions upon post-transcriptional and post-translational modifications. Because restoring tumor suppressor functions remains the most effective and selective approach to induce apoptosis in cancer, the dissection of mechanisms of tumor suppressor inactivation is advisable in order to further augment targeted strategies. This review will summarize the role of tumor suppressors in chronic lymphocytic leukemia and attempt to describe how tumor suppressors can represent new hopes in our arsenal against chronic lymphocytic leukemia (CLL).

## 1. Introduction

Chronic lymphocytic leukemia (CLL) is the most common adult leukemia in the western world accounting for 25% of all adult leukemias [1]. In these countries, the incidence of CLL is almost 5 new cases per 100,000 adults, with a peak of incidence in people older than 70 years [2]. CLL is a cancer of mature B lymphocytes that are clonally expanded and accumulated in the blood, bone marrow, and lymphoid tissues. The presence on peripheral blood of ≥5000 lymphocytes/μL with the typical immunophenotypic co-expression of T cell marker CD5 and B cell surface antigens CD19, CD20, and CD23 with low levels of monoclonal immunoglobulin defines CLL diagnosis according to the international working group of CLL [3,4]. The clinical course of CLL is variable, as extensively reviewed [5]. Chemoimmunotherapy was recognized as the standard of treatment for young patients with CLL (below 65 years of age) who can benefit from fludarabine and cyclophosphamide with anti-CD20 monoclonal antibody. More recently, the inhibitor of Bruton’s tyrosine kinase; ibrutinib [6]; the inhibitor of **Phosphoinositide 3-kinases** (PI3-kinase) catalytic subunit delta (idelalisib) [7]; and the inhibitor of **B-cell lymphoma 2** (Bcl-2), venetoclax [8], have irrupted into the clinical arena, offering the change for a selective, chemo-free approach of cure. With the introduction of these target molecules, it is crucial to well-stratify CLL patients with the aim of identifying those who can really benefit from a chemotherapy approach (e.g., *immunoglobulin heavy chain variable region gene IGHV*-mutated) and who cannot (e.g., tumor protein p53, *TP53*-mutated). *TP53*-mutated/deleted CLL patients are currently approached with novel drugs due to the innate resistance to chemotherapy that only provide a change to further select more resistant and aggressive clones [9].

However, the aim of CLL eradication remains a challenging issue, and the need of perfect combination of the drugs and/or new targets is mandatory. Originally, tumor suppressors were described as major players in cancer that act when lost. Currently, the involvement of tumor suppressors in cancer is much more complex. Besides the lack of tumor suppressors because of mutations/deletions, it is clear that some tumor suppressors are also impaired when functionally inactive through epigenetic and/or post-translational mechanisms [10,11]. Various genetically wild-type tumor suppressors appeared inactive due to variations in expression, functionality, and/or cellular compartmentalization.

The portrait of the CLL genome has been taken with great detail, in either coding or non-coding compartments [12,13,14]. Overall, these great efforts have pointed out that the number of copy number aberrations is low, with a range of 0–2 lesions per patient, suggesting that complex mechanisms may promote CLL development and maintenance, besides genetics. Although on one hand such a scenario may impose further investigations to dissect mechanisms of CLL pathogenesis, on the other it could revive new therapeutic options. This review summarizes the role of tumor suppressors in CLL, focusing on both deleted/mutated tumor suppressors and on functionally inactive ones. In the first part of this review, we describe the genetic impairment of tumor suppressors in CLL through mutations and deletions, as found for various *TP53*, ataxia telangiectasia mutated (*ATM*), and others. Next, we describe tumor suppressors that are functionally inactivated, including modifications at the transcriptional and protein levels.

## 2. Mechanisms of Tumor Suppressor Inactivation in CLL: Genetic Loss of Function

**Figure 1:**
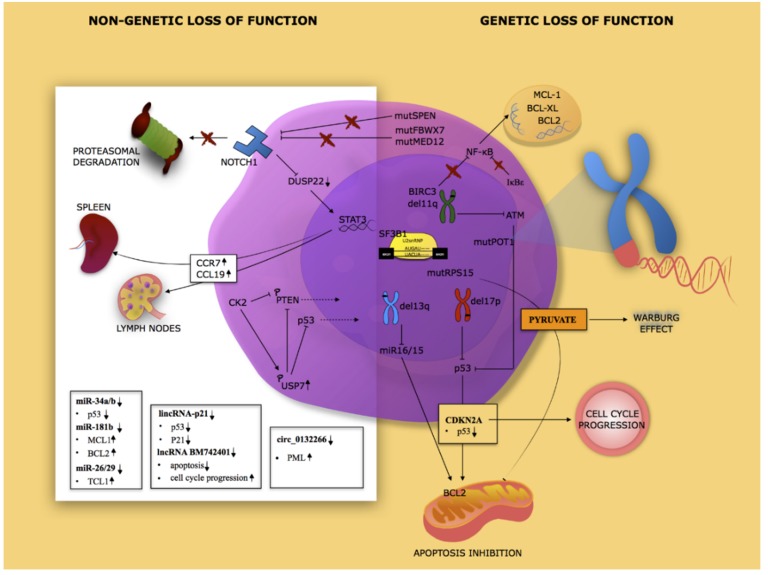
Landscape of the main tumor suppressors inactivated in chronic lymphocytic leukemia. Tumor suppressors are inactivated in chronic lymphocytic leukemia (CLL) patients by several mechanisms. Genetic lesions involving *TP53*, ataxia telangiectasia mutated (*ATM*), protection of telomers 1 (*POT1*), *BIRC*, and *NOTCH1*. Post-translational inactivation includes, for example, PTEN and p53. Post transcriptional mechanisms include microRNA 15/16, long non-coding RNA (lncRNA), and circular RNA (circRNA).

In this section, we discuss the most frequently inactive tumor suppressors through deletion or mutational inactivation of the genes (Figure 1).

### 2.1. Deletion of 13q14 Locus

Deletion 13q14 is the most common chromosomal abnormality detected in CLL (≈60% of cases), followed by 11q and 17p deletions (18% and 8% of CLL cases respectively), and by trisomy 12 (12–16% of cases) (***National Comprehensive Cancer Network*** NCCN, Version 2.2020). This deletion is generally found in heterozygosity and spans through a variable region of chromosome 13 among patients [15]. The most common region targets the tumor suppressor locus **deleted in lymphocytic leukemia 2** *DLEU2/MIR15A/MIR16A* [16]. In a portion of patients, this deletion has been found as a biallelic deletion; however, no clear differences between mono- and biallelic deletions were found in CLL patients [15]. Besides the presence of such deletion, these genes are often downmodulated in CLL patients, suggesting the presence of additional mechanisms of regulation with potentially relevant implications in the pathogenesis of CLL. Clinically, the presence of this deletion is associated with a favorable prognosis. Experimentally, few murine models have narrowed the pathogenetic role of this deletion to miR-15a-miR16-1 [17]. In normal cells, miR-15a and miR-16-1 inhibit the expression of B cell CLL/B-cell lymphoma 2 (*BCL2*), as well as the cyclins Cyclin D1 (*CCND1)* and *CCND3*, and cyclin-dependent kinase 6 (*CDK6)* [18]. In this respect, it is worth noting that the main cause of Bcl2 overexpression in CLL is the loss of *miR-15a* and *miR-16-1* consequential to 13q14 deletion, whereas de-repression of BCL2 is due to the lack of microRNA-mRNA interaction [19]. Furthermore, recent reports have also elucidated the link between *miR-15a*/*miR-16-1* and *TP53* expression. An inverse correlation between expression of the above-mentioned microRNAs and p53 levels has been established both in cell lines and in CLL patients owning del13q. A binding site for *miR-15a* and *miR-16-1* has been identified inside the 3’-untranslated region of TP53 [20,21]. Additionally, 13q14del CLL express low levels of telomere-specific reverse transcriptase activity (TERT). This is partially explained by the fact that p53 directly represses the transcription of *TERT* [22]. Patients harboring 13q14 deletion as a sole abnormality have a favorable clinical outcome, with the longest median survival among all CLL patients (NCCN, Version 2.2020). This kind of indolent subtype of CLL can be considered the intriguing result of the balance between oncogene and oncosuppressor activation originated from a unique chromosomal deletion, mutation, or epigenetic alteration ending in the reduced expression of the primum movens oncosuppressor *miR-15a*/*miR-16-1*.

### 2.2. TP53

*TP53* is one of the most frequently mutated tumor suppressors in cancers [23]. Mutations of the TP53 gene and deletion of the chromosome 17p13 (17p-) impair the function of the p53 tumor suppressor and identify a very high risk CLL group of patients [24]. *TP53* aberrations occur in almost 10% of CLL patients, but the frequencies further increase in resistant and progressing CLL patients, as has been extensively reviewed [18]. Due to the role of p53 in the modulation of apoptosis, lack of p53 is undoubtedly associated with a more aggressive, instable, and chemo-resistant form of CLL. It is worth to noting that *TP53* (minor)-subclones are easily selected by chemotherapy, allowing it to change from a more indolent CLL form into a chemo-refractory one [9]. As a consequence, investigation of *TP53* mutations/deletion is a mandatory approach before the enrollment of a CLL patient to therapy [25]. Lastly, *TP53* aberrations were shown to play a role in the progression of CLL towards Richter syndrome (RS) [26].

### 2.3. ATM/del(11q)

Deletions in the 11q22-23 locus (commonly referred as 11q-) are recognized in a range between 11% and 18% in non-biased CLL cohort, but it increases with the Binet stage. This aberration is generally associated with the IGHV unmutated phenotype and is expressed in heterozygosity. The deletion of this portion of chromosome 11 is associated with the loss of ataxia telangiectasia mutated (*ATM*) gene. ATM is a serine/threonine protein kinase acting as a tumor suppressor gene. It dictates cellular responses during DNA damage [27]. The lack of ATM is associated with the development of genomic instability and therefore a more aggressive phenotype. Del(11q), often associated with extensive lymphadenopathy, disease progression, and shorter median survival (≈40% of cases alive at 10 years), is an adverse factor that identifies a group of patients with intermediate-risk disease.

### 2.4. SF3B1 Mutations

*Splicing factor 3b subunit 1 (SF3B1)* mutations are another commonly identified somatic mutation in CLL and are associated with poor clinical outcome [28]. In particular, the majority of mutations in SF3B1 have been identified inside the conserved C-terminal domain. SF3B1 is an essential component of the splicing machinery, where it is involved in the removal of introns from precursor messenger RNA (pre-mRNA). Due to this role, *SF3B1* mutations affect the splicing of pre-mRNA. Consistent with this, several cases of altered splicing have been detected in CLL with SF3B1 mutations. More in depth, it has been observed that mutations of *SF3B1* do not impair splicing in an aspecific manner, but it seems to affect only a few specific targets, including the forkhead box P1 (FOXP1) a forkhead transcription factor (Figure 1) [29].

### 2.5. RPS15 Mutations

**40S ribosomal protein S15** is ribosomal proteins encoded by the *RPS15* gene, involved in the regulation of the MDM2–p53 axis and proteosomal degradation of p53 [30]. RPS15 mutations have been identified in newly diagnosed, untreated CLL, but also in 20% of relapsed patients [31]. Unfortunately, the molecular mechanisms between the RPS15 mutations and CLL pathogenesis are unclear. Using the 293t cellular model and the CLL cell line MEC-1, Bretones and colleagues demonstrated that RPS15 mutations affect the stability of RPS15 protein. In addition, some RPS15 mutations are involved in ribosome biogenesis defects. Furthermore, it has been observed that the pathways perturbed by RPS15 mutations are different according to the cellular model used, including metabolic reprogramming toward the Warburg effect (Figure 1) [32].

### 2.6. BIRC3 Mutations

*BIRC3* (baculoviral IAP repeat containing 3) is a recurrently mutated gene in CLL [33]. Both monoallelic deletions and/or truncating mutations have been reported. These abnormalities impact on BIRC3 functions as the ubiquitin ligase of NIK, an activator of the nuclear factor kappa-light-chain-enhancer of activated B cells (NF-κB) pathway, therefore ultimately favoring NF-κB activation [34]. As a consequence of enhanced p65 activity, an increase in mRNA expression of Bcl-2, Bcl-XL, and the Induced myeloid leukemia cell differentiation protein (Mcl-1) have been observed. Importantly, these findings suggest a possible different sensitivity to treatment with agents targeting Bcl-2 and/or Bcl-XL.

### 2.7. NFKBIE Mutations

The Nuclear factor of kappa light polypeptide gene enhancer in B-cells inhibitor, epsilon, *also known as NFKBIE* gene, which encodes for the Inhibitor of kappa B epsilon IκBε, a negative feedback regulator of NF-κB, is found mutated in 1–3% of CLL patients [35,36,37]. NFKBIE is associated with the reduction of IκBε protein, which in turn favors p65-NF-κB activation. Moreover, IκBε loss was associated with increased B cell proliferation and survival upon immune system activation in mouse. In particular, it was demonstrated that B cells purified from mixed splenocytes collected from IκBε^−/−^ mice and stimulated with Lipopolysaccharides (LPS) displayed increased expansion compared to wild type B cells. In accordance with the inhibitory activity of IκBε against NF-κB, IκBε^−/−^ B cells increased expression of NF-κB target genes. More specifically, LPS stimulation in IκBε^−/−^ B cells induced interleukin IL-6 expression, which in turn was associated with RelA hyperactivation [38].

### 2.8. POT1 Mutations

Telomeric changes are recognized as occurring in CLL, playing a prognostic role [39]. Among the complex family of genes involved in telomere formation [40], protection of telomers 1 (*POT1*) was found mutated in 3% of CLL patients and also in few cases of RS [41,42]. The lack of *POT1* was associated with the development of telomeric and chromosomal abnormalities, caused by the inability of the cells to engage a DNA damage response [41]. POT1 is a component of the telomerase ribonucleoprotein (RNP), essential for the replication and the regulation of telomere termini. Interestingly, using immunofluorescence microscopy analysis, Ramsay and colleagues established that mutated POT1 promotes its co-localization with the telomere-binding proteins (TRF). Mutated *POT1* induces dysfunctional telomeric phenotype characterized by elongated and unprotected telomere ends that are responsible for chromosomal alterations. 

### 2.9. RARRES3 Deletion

The deletion of the retinoic acid receptor responder 3 gene (*RARRES3*) is associated with 11q22 deletion. RARRES3 controls cell growth in a manner closed to retinoid acid [43]. RARRES3 expression appeared mostly downmodulated in advanced CLL stages and appeared involved in CLL progression.

### 2.10. Rare Events

Various other tumor suppressor genes have been found mutated in CLL. The cell cycle inhibitors *CDKN1B*, cyclin-dependent kinase inhibitor 1B (p27^Kip1^), and *CDKN2A*, coding for p16INK4a, an inhibitor of cell cycle, as well as p14arf, the negative regulator of MDM2, were found mutated in CLL [12]. Despite being rarely mutated in CLL, the analysis of *CDKN2A* locus revealed that CDKN2A losses are commonly detectable in RS [44]. **F-box/WD repeat-containing protein 7**, *FBXW7*, mediator complex subunit 12 (*MED12*), and spen family transcriptional repressor (*SPEN*), three tumor suppressors associated with the regulation of the NOTCH1 pathway, were also identified as being mutated with low frequency in CLL. In particular, *FBXW7* and *MED12* mutations prevent proteasomal degradation of NOTCH1, whereas SPEN interferes with NOTCH1 signaling [45,46,47]. Interestingly, the homozygous deletion of the *SPEN* gene was found at RS diagnosis [48]. The tumor suppressors Paired box protein (*Pax5*), *protein-tyrosine phosphatase 1B (PTPN1)*, and chromodomain helicase DNA binding protein 2 (*CHD2*) were also identified among mutated genes in CLL [49].

## 3. Mechanisms of Tumor Suppressor Inactivation in CLL: Functional Inactivation through Post-Translational Mechanisms

This section will focus on genetically wild-type tumor suppressors whose functions are affected at a post-translational level (Figure 1).

### 3.1. PTEN

The tumor suppressor *PTEN* is one of the most frequently mutated/deleted tumor suppressors in cancer [50]. Besides genetic impairment, it is worth noting that *PTEN* inactivation is also favored by post-translational modifications and/or changes in protein compartmentalization [51,52,53]. In particular, PTEN was shown to lose various tumor suppressive functions when aberrantly delocalized in the cells, through changes in mono-ubiquitination [54,55] and sumoylation [56]. Although cytoplasmic/membrane-bound PTEN can modulate PI3-kinase signaling, nuclear PTEN has been found to play a tumor suppressive role by modulating genomic stability, proliferation, and survival through the interaction with other targets [57]. Recently, we demonstrated that Ubiquitin-specific-processing protease 7 (USP7) is aberrantly expressed in CLL, favoring the deubiquitination of PTEN [58]. More precisely, CLL samples mostly expressed the serine-18 USP7 isoform that can be regulated by casein kinase II-mediated phosphorylation. Consequently, PTEN is deubiquitinated, inducing the loss of its nuclear compartmentalization. Treatment with USP7 inhibitor P5091 promotes PTEN re-localization into the nucleus. PTEN was also shown to be functionally inactivated in CLL through tail phosphorylation by casein kinase II (CK2) [59]. In particular, CK2 and PTEN physically interact, and CK2 mediates its phosphorylation at the C-tail residues, leading to blockade of PTEN phosphatase activity. Pten inactivation is essential for maintenance of CLL cell viability. Therefore, inhibition of CK2 resulted in promoting CLL apoptosis [60,61], as reviewed and described in other cancers [59,62].

### 3.2. p53

As we have previously discussed, genetic aberrations of the tumor suppressor *TP53* are relevant in CLL pathogenesis [63]. Parallel to genetic modifications, it is known that p53 functions can be modulated by various post-translational modifications [64]. In particular, mono- and poly-ubiquitination are known to affect the localization and/or the stability of the tumor suppressor p53. In particular p53 de-ubiquitination by USP7 was shown to modulate its nuclear-cytoplasmic shuttling. Due to the ability of p53 to modulate the expression of various pro-apoptotic genes, the removal of p53 from the nucleus is undoubtedly a mechanism of tumor suppressor inactivation. As we discussed before, USP7 was shown to be over-expressed in various CLL patients. Besides its ability to modulate PTEN compartmentalization, USP7 aberrant expression was investigated for its ability to modulate the localization and stability of p53.

### 3.3. Puma

Together with Noxa and Bin, p53 upregulated modulator of apoptosis (Puma) is a BH3 protein controlled by p53 that is able to contribute to the p53-mediated apoptosis [65]. Besides its activity as a transcriptional factor, p53 has also been shown to regulate apoptosis through a non-transcriptional process. In particular, p53 protein binds to the antiapoptotic Bcl-2 family proteins at the mitochondria, favoring apoptosis [66,67,68]. Such a complex process was also shown to be activated in CLL cells [69], allowing them affect apoptosis responses to chemotherapy. Other authors have also demonstrated that Puma levels may have a prognostic role in CLL. In more detail, it has been described that Puma upregulation after treatment with fludarabine was strictly related not only to p53 but also to *IGVH* mutation status. Further study is necessary to link p53-mediated Puma upregulation and *IGVH* mutation status in CLL [70,71].

## 4. Mechanisms of Tumor Suppressor Inactivation in CLL: Epigenetic

Genome and epigenome are intimately connected in cancer development, and the interplay of genomic and epigenomic factors plays a major role in CLL pathogenesis as well. In a recent analysis of the epigenomic landscape, various aberrations of the epigenome have emerged as markers in CLL pathogenesis [72,73]. However, it is also worth noting that methylation is a process that may occur both in normal and pathological hemopoiesis. In this respect, the methylome of normal B cell maturation and of CLL was solved and compared [74]. In this impressive work, the panel of hypermethylated genes from the CLL dataset appeared closed to those observed during normal B cell maturation, suggesting the methylation may not be considered as a driving force in CLL tumorigenesis. However, with that in mind, an aberrant methylation programming was also observed in CLL, affecting various genes, including **Activator protein 1** (*AP-1*), Early B-Cell Factor 1 (*EBF1*), and Runt-related transcription factor 3 (*RUNX3*) (Figure 1).

### 4.1. Promoters’ Hypermethylation

The methylation of a gene promoter allows for the modulation of the expression of various genes. Notably, such a mechanism of gene regulation has been linked to CLL pathogenesis [75], although some concerns on this mechanism should be raised, as previously described [74]. Moreover, recent evidence also highlights the fact that methylome changes in response to chemoimmunotherapy treatments. In this respect, epigenetic evolution has been observed in relapsed patients compared to pre-treated patients [76]. However, various tumor suppressor genes have been described as being highly methylated, with a correlation to disease stages and/or prognosis, including the Krüppel-like factor 4 (*KLF4*) gene [77], p15 [78], and Patched (*PTCH*) [79]. The tumor suppressor *PHLPP1* (phosphatase PH domain leucin-rich repeat protein phosphatase) is an important tumor suppressor that directly regulates the Protein kinase B (PKB), also known as **Akt** kinase [80]. Alteration in PHLPP1 mRNA levels have been observed in a great portion of CLL patients where Akt signaling was further augmented [81]. PHLPP1 downmodulation in CLL was associated with methylation at the end of exon 1 [82]. Recent findings suggest a possible role of *NOTCH1* mutations in affecting methylation promoter status. Specifically, Arruga and colleagues, using *Mec-1/NOTCH1* knock-out KO and expressing wild-type or mutant NICD, demonstrated that NOTCH1 PEST-domain mutations regulated Dual specificity protein phosphatase (*DUSP22*) promoter methylation and consequently its expression. DUSP22 is a phosphatase able to modulate **mitogen-activated protein kinase** (MAPK) family members and inactivate **Signal transducer and activator of transcription 3** (STAT3). Accordingly, Mec-1 expressing NOTCH1 PEST-domain mutation showed methylation of the DUSP22 promoter and high levels of STAT3, which in turn induces CCR7 (C-C chemokine receptor type 7) expression and CCL19 (Chemokine (C-C motif) ligand 19)-driven chemotaxis [83].

### 4.2. Intronic Polyadenylation

Parallel to genetic aberrations involving genomic DNA, meaning DNA mutations and gross chromosomal aberrations, abnormalities in RNA processing may promote tumorigenesis as well [84]. Among these processes involved in RNA maturation, intronic polyadenylation (IPA) allows for the generation of truncated mRNA molecules the modify transcriptome, even if originating from the same wild-type gene. Therefore, IPA was shown to favor the maturation of cells from the immune system. Recently, IPA was shown to play a pivotal role in CLL, particularly as a mechanism of inactivation of various tumor suppressor, irrespective of their genetic status [85].

## 5. Mechanisms of Tumor Suppressor Inactivation in CLL: MicroRNA (miRNA), Long Non-Coding RNA (lncRNA), and CircularRNA

The interest of the scientific community on the genetic “dark matter” of our genome has emerged recently. Indeed, only in 2012 and thanks to extraordinary effort of the Encode consortium has there been a clear demonstration that the vast majority of our genome is transcribed in long non-coding RNA molecules (lncRNAs, > 200 nts), including also pseudogenes and microRNAs (miRNAs) [86]. More recently, another abundant class of RNA regulatory transcript has been described—circular RNAs (circRNAs). This category of RNA molecules is particular abundant, as circRNAs can derive from both coding and non-coding RNA (ncRNA) transcripts [87]. Extensive whole genomic sequencing of CLL primary samples have raised the relevance of alteration in the non-coding sequences of CLL genomes [14].

### 5.1. miRNA

A pivotal contribution on this topic has been provided by Croce’s group. In 2002, they identified in the chromosome 13q14 region, a portion frequently deleted in B-CLL, two miRNAs, miR-15, and miR16 that act as oncosuppressors in this malignancy. This miRNA cluster regulates, at the post-transcriptional level, different oncogenes including the myeloid cell leukemia 1 (MCL1) and the B cell CLL/B cell lymphoma 2 (BCL2), which exert an oncogenic function in this hematological disorder [88]. It worth noting that another oncosuppressor miRNA cluster, miR-34a/b, is located in a different deleted region, the 11q. This cluster controls zeta-associated protein (ZAP70) levels, a critical prognostic marker for CLL patients [20]. Notably, both miR34a/b and miR-15/16 expression is under the direct transcriptional regulation of p53 [20,89], supporting the relevance of these clusters in cancer pathogenesis. Moreover, the 11q region is also frequently hypermethylated in CLL, defining an additional mechanism for the inactivation of miR34a/b in this disease [90]. Furthermore, miR-181b is commonly decreased in CLL samples with progressive disease. miR-181b regulates **T-cell leukemia/lymphoma protein 1** (*TCL1*), which is involved in the aggressive form of CLL. Moreover, miR-181b-responsive elements are observed in the 3’-UTR (untranslated regions) of the anti-apoptotic genes *BCL2* and *MCL1*. Accordingly, miR-181b down-regulation inversely correlates with the expression of these genes, which are up-regulated in CLL patients with progressive disease. Due the important role of miR-181b in the regulation of these three key genes, it was considered as a unique biomarker for CLL monitoring [91,92,93].

Another miRNA with tumor suppressive activity in the CLL context is miR-34a (a microRNA involved in the p53 pathway). This miRNA is significantly down-modulated in several cases of refractory disease. Moreover, very low levels of miR-34a are observed in cases of the 17p deletion end/or TP53 mutation [94]. Among miRNAs deregulated in CLL, the miR-29 family has been described as down-regulated in aggressive B-CLL. miR-29 strongly regulates TCL1 and plays an important role in the pathogenesis and aggressiveness of tumorigenic cells [95]. Finally, the role of miR-26 in cancer is more controversial, but its delivery in the Eμ-TCL1 transgenic (TCL1-tg) model of CLL is anti-leukemic [96]. Lastly, it is worth noting that one of the most frequent non-coding lesions include mutations in the 3’-UTR of NOTCH1 and mutations in a specific enhancer of chromosome 9q13.

### 5.2. LncRNAs

LncRNAs represent another heterogeneous family of ncRNA molecules that play a role in CLL malignancy [97]. Additionally, in this context, p53 modulates the expression of different lncRNAs, thus revealing a p53-lncRNA tumor suppressor signature that characterizes CLL pathogenesis [97,98]. Among these, the lncRNAs nuclear enriched abundant transcript 1 (NEAT1) and long-intergenic ncRNA p21 (lincRNA-p21) are involved in the DNA damage response in CLL and modulate apoptosis only in the presence of a functional p53. Because lincRNAp21 modulates in cys p21, its expression directly correlates with p21 levels in CLL patients [99]. Moreover, circulating lincRNA-p21 levels decrease in CLL patients, especially in advanced disease [100]. Interestingly, the frequent deleted region 13q14.3 also includes two lncRNAs, DLEU1 and a variant DLEU2. In contrast to the DLEU2 region containing the miR15/16 cluster, which is down-modulated in CLL, the lncRNA *DLEU1* and *DLEU2/Alt1* loci are demethylated and thus upregulated in CLL, defining a complex ncRNA interplay in this chromosomal region [101]. Recently, it has also been shown that the tumor suppressor lncRNA BM742401 is inactivated by DNA methylation in CLL. Interestingly, BM742401 methylation correlates with a high Rai stage. More specifically, BM742401 over-expression in CLL cell lines induces cell cycle arrest and enhances cell apoptosis through caspase-9 [102].

### 5.3. CircRNAs

CircRNAs originate from a process called back-splicing, which involves both coding and ncRNA transcripts. Mechanistically, circRNAs modulate gene function at the transcriptional and post-transcriptional level [103]. Recent evidence supports a critical role of circRNA in tumorigenesis [104], and their implications in CLL are now emerging. For instance, circ_0132266, a circRNA down-modulated in CLL, acts as a miRNA sponge for promyelocytic leukemia protein (PML), and thus modulates cell viability [105].

## 6. Therapeutic Implications

The role of tumor suppressors in cancer pathogenesis and maintenance has dramatically changed from essential and untouchable actors to potentially selective bullets able to promote cancer cell suicide, if turned on. It is worth mentioning that the most powerful pro-apoptotic signal in cancer is the reactivation of tumor suppressors themselves [106,107]. For instance, the restoration of p53 expression in mouse was shown as the most efficient approach to force cancer exhaustion [107]. Only few years ago, however, tumor suppressors did not appear targetable, unless through indirect approaches. For example, in the case of PTEN deletion, one could administrate PI3-kinase inhibitors to block the increased phosphatidylinositol-3,4,5-triphosphate (PIP3) signaling; however, they would lack the possibility of targeting the PI3K-independent functions of PTEN.

**Figure 2:**
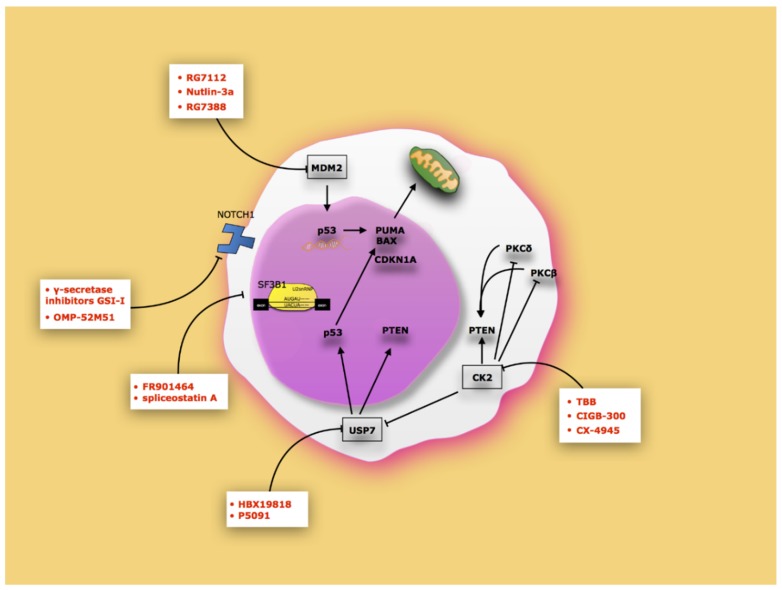
Therapeutic approaches to reactivate tumor suppressor in CLL. The schema shows the main inhibitors that can restore tumor suppressor function.

Luckily, the involvement of tumor suppressors in cancer is substantially changed. Tumor suppressors are no longer important in cancer pathogenesis only when mutated or deleted, but are also important when functionally inactive by changes in levels, cellular localization, or by post-translational modifications [10,108,109]. In such a respect, various functionally inactive tumor suppressors can be potentially turned on. In the CLL context, clear examples of these opportunities are p53 reactivation with the Mdm2 inhibitors [110,111,112] (Figure 2). Mdm2 inhibitors such as RG7112, RG7388, and nutlin-3a induce non-genotoxic activation of p53, thus stabilizing and transcriptionally activating it. In the same way, modulation of USP7 activity by HBX19818 and P5091 inhibitors can restore both p53 and PTEN activity [58,113]. Inhibition of CK2 could be used to reactivate PTEN. In particular, CK2 inhibitors induce CLL apoptosis by reducing the activity of **Protein Kinase C** (PKCβ) and PKCδ, two downstream factors of PI3K, and preventing PTEN phosphorylation, thus stabilizing PTEN. CK2 inhibition also reduces USP7 activity, which in turn is responsible for PTEN nuclear loss of function [114].

It is also possible to speculate on the use of drugs that can prevent the export of pre-mRNA aberrant events generated by SF3B1 mutations to the cytoplasm. In this regard, the use of FR901464 or spliceostatin A inhibit in vitro splicing generated by SF3B mutation [115]. Finally, the inhibition of NOTCH1 on the basis of the use of molecules that block its proteolytic cut (γ-secretase inhibitors (GSIs)) or humanized antibodies (OMP-52M51) may also be re-activated by tumor suppressors inhibited by its activity. Further analyses are, however, advisable to achieve a better comprehension of the role of functional inactivation of tumor suppressors in CLL and on the strategies to reactivate them.

## 7. Conclusions

The massive parallel sequencing of coding and non-coding genome of hundreds of CLL samples were reported, together with methylome and comparative analyses with normal B cell development. Such analyses have raised some important points: (i) the classical aberrations on coding genomic DNA are only one aspect of CLL pathogenesis, (ii) changes in non-coding DNA are equally relevant in CLL pathogenesis, (iii) DNA methylation is a more complex modification that must be reconsidered in CLL as well as in normal B cell development, and (iv) various tumor suppressors are functionally impaired irrespective of to their genetic status. Consequently, if on one hand mechanisms of CLL development and maintenance appear much more complex than originally stated, on the other hand these new investigations have raised new options from the therapeutic standpoint and hope to achieve CLL eradication.
